# Verbal Abuse Related to Self-Esteem Damage and Unjust Blame Harms Mental Health and Social Interaction in College Population

**DOI:** 10.1038/s41598-019-42199-6

**Published:** 2019-04-04

**Authors:** Je-Yeon Yun, Geumsook Shim, Bumseok Jeong

**Affiliations:** 1 Seoul National University Hospital, Seoul, Republic of Korea; 2 Yeongeon Student Support Center, Seoul National University College of Medicine, Seoul, Republic of Korea; 3 KAIST Clinic Pappalardo Center, Korea Advanced Institute of Science and Technology (KAIST), Daejeon, Korea; 4 Graduate School of Medical Science and Engineering, Korea Advanced Institute of Science and Technology (KAIST), Daejeon, Korea; 5 KAIST Institute for Health Science and Technology, KAIST, Daejeon, Korea

## Abstract

Verbal abuse is an emotional abuse intended to inflict intense humiliation-denigration-fear as perceived by exposed person. Network-based approaches have been applied to explore the integrative-segregated patterns of associations among the psychological features and external stimuli for diverse populations; few studies reported for verbal abuse effects in college population. Self-reporting measurements acquired form 5,616 college students were used for network analyses. Escalating cascades of verbal abuse from differential sources (parents, peers, or supervisors; network 1) and directed associations among verbal abuse severity-psychopathology-social interaction (network 2) were estimated using the directed acyclic graphs. Principal connectors of verbal abuse–psychopathology–social interaction were shown using the graph theory metrics calculated from the intra-individual covariance networks (network 3). Directed propagating patterns of verbal abuse phenomena differed by source (network 1). Severe peer-related verbal abuse affected psychomotor changes and influenced irritability (network 2). Verbal abuse of self-esteem damage and unjust blame served as connectors in the verbal abuse-psychopathology-social interaction; influence of smartphone overuse-related distress was stronger in cases with more severe verbal abuse (network 3). Verbal abuse that damages self-esteem and conveys unjust blame harms mental health and social interaction for college population.

## Introduction

Verbal abuse is a form of emotional abuse intended to inflict intense humiliation, denigration, or extreme fear, as perceived by the victimised person^1^. Perceived parental verbal abuse in childhood and peer-related verbal abuse in adolescence have been associated with a risk of depressive mood, anxiety, anger-hostility, suicidality, dissociation, or drug use in young adulthood^2–6^. Moreover, experience of perceived verbal abuse has been associated with changed patterns of brain maturation, including the reduced structural integrity of brain white matter bundles^7^, compromised brain resting state functional connectivity^8,9^, and decreased brain grey matter volumes in regions responsible for sensory processing, emotional regulation, and social interaction-related cognitive functioning such as language and memory. All of the above factors have been suggested to reflect the neural underpinning of the psychopathology^10–16^. Further, perceived verbal abuse in adulthood in relation to intimate partner violence and workplace mistreatment also affects brain morphology and undermines mental health^17,18^. However, unlike the extreme clinical syndromes developing after trauma, such as post-traumatic stress disorder^19,20^, few studies have explored interactions among perceived, verbal abuse-psychopathology-social interaction patterns in young adult populations.

Using network-based approaches, integrative as well as segregated patterns of interactions among the psychopathology, cognitive functioning, and perceived external stimuli have been explored in various populations^19,21–23^. In such networks, each psychological feature is considered to be a node; these nodes are connected with edges that represent strengths (with or without directionalities) of relationships among the nodes that collectively comprise the network. Depending on the data characteristics and the aims of study, several formats of networks are available; the directed acyclic network (DAG; a directed and group-wise Bayesian network)^22,24^, a Gaussian graphical model (an undirected, partial correlation network in which edges represent group-wise relationships between ordinal or continuous variables)^25–27^, an Ising model (an undirected network estimating group-wise relationships among the dichotomous variables)^28,29^, and an intra-individual covariance network (an undirected network that describes inter-connectedness between psychological constructs within each participant)^23^.

To the best of the authors’ knowledge, this study is the first network-based approach that explored the escalating patterns of verbal abuse according to differential sources in addition to the directional associations between the severity of perceived verbal abuse versus the psychopathology and social interaction pattern. To explore the escalating cascades of perceived verbal abuse (*Network 1*) and the directed relationships among the perceived verbal abuse–psychopathology–social interaction patterns (*Network 2*) in college population, we used a dataset of self-reporting measurements acquired form 5,616 undergraduate and graduate students and retrieved the directed acyclic graphs (DAGs; the graphical structures of the Bayesian networks). The DAG defines probabilistic dependencies [shown as directional edges; based on the Markov property of Bayesian networks (=a direct dependence of every nodes only on their parental nodes)] among the components (visualized as nodes) of Bayesian network^30^; alike in previous studies^19,21,22^ that successfully uncovered the directed associations or causal relations among the diverse psychological features, the current study applied a score-based heuristic local search method of ‘hill-climbing’ as implemented in an R package *bnlearn*^30^. With hill-climbing algorithm, procedure for learning the graphical structure of Bayesian network (=DAG) starts from the initial solution of network structure and traverse the search space across the nodes by repeated attempts of network structure change - add, delete, or reverse of the directional edges that connect specific nodes with their neighboring nodes - to only reflect the changes of network structure (=edge) that greatly improves the fit of network to dataset^31^. Meanwhile, to keep the DAG from being trapped in local optima during the middle of hill-climbing-based searches, consensus-based solution of DAG is finally retrieved from the several runs of greedy search trials (each initiated from randomly chosen nodes) using hill-climbing; after learning the global probability distribution [=factorization of the joint probability distribution] of network, parameters of the local probability distributions for each nodes (conditional on the learned network structure) are estimated^30^. Further, to identify the principal components among the inter-variable covariation [=degree of similarity between two clinical variables in terms of the deviation from mean values (calculated from the whole participants) for each variable within an individual] of perceived verbal abuse–psychopathology–social interaction at the individual level, we retrieved global and local graph metrics^32^ from the intra-individual covariance network^33^ employing self-reporting measures for the same 5,616 students.

In this study, we first hypothesised that escalation of verbal abuse severity might differ by source (parents, peers, or supervisors). Also, we hypothesised that an influence cascade would emerge featuring the patterns of social interaction, the severity of perceived verbal abuse, and the intensity of psychological suffering (depressive mood, anxiety, substance abuse and inefficient cognitive style in daily living). Notably, previous studies found that poor perceived self-efficacy and perceived injustice triggered depression^34–37^, generalised and social anxiety^37–41^, addiction to alcohol and smartphone use^42,43^, and adult ADHD-like symptoms^44–46^. Certain verbal abuse components (attacks on self-efficacy or perceived injustice) were considered as candidate hubs connecting different components of the perceived, verbal abuse-psychopathology-social interaction patterns; thus, these were useful shortcuts.

## Methods

### Study population

We used de-identified responses for self-reporting questionnaires (please refer to the ‘Measures’ section) completed during annual healthcare screening of 5,616 undergraduate and graduate students between April 2014 and February 2015 at the KAIST Clinic (https://clinic.kaist.ac.kr). Participants ranged from 18 to 49 years of age (mean = 23.3 years, S.D. = 4.0 years). We evaluated 4,498 males (80.1%) and 1,118 females (19.9%). The Institutional Review Board at KAIST approved the current study (IRB approval no. KH-2012–16), and written informed consent was obtained from all subjects after the procedures had been fully explained. All procedures were performed in accordance with the ethical standards of the KAIST IRB on human experimentation and the Helsinki Declaration of 1975, as revised in 2008.

### Measures

To measure the psychopathology of depressive mood (using the patient health questionnaire-9 (PHQ-9))^47–49^, anxiety (by applying the generalised anxiety disorder 7-item (GAD-7))^50–52^, substance abuse (alcohol; using the CAGE questionnaire)^53–55^, cognitive style of daily living (by applying the adult attention-deficit/hyperactivity disorder (ADHD) self-report scale (ASRS-v.1.1))^44–46^, as well as social interaction patterns [of non-confrontational coping, including the anxiety-fear-avoidance for social situation^56–59^ (the Liebowitz social anxiety scale (LSAS)) and preference for non-face-to-face social interaction combined with smartphone overuse^60–62^ (the smartphone addiction scale (SAS))], that have been associated with perceived verbal abuse (by applying the verbal abuse questionnaire (VAQ)), we applied several self-reporting questionnaire listed below.

#### Depressive mood: Patient Health Questionnaire-9 (PHQ-9)

The PHQ-9 is a nine-item module assessing the severity of depressive symptoms, including low-level interest or pleasure, feeling down and hopeless, trouble sleeping, tiredness or having little energy, poor appetite/overeating, guilt, trouble concentrating, moving slowly/restlessness, and suicidal thoughts^63^. Here, the item-level responses for the Korean-validated version of PHQ-9^64^ served as the nine depressive mood components (nodes) for network analyses^26^.

#### Anxiety: Generalised Anxiety Disorder 7-item (GAD-7)

The GAD-7 instrument features seven items exploring nervousness, uncontrollable worry, worrying about different things, trouble relaxing, restlessness, irritability, and the fear that something awful might happen; respondents report the severity of each symptom using a 4-point Likert scale [from 0 = ‘not at all’ to 3 = ‘nearly every day’]^65^. Here, item-level responses for the Korean-validated version of GAD-7^66^ served as anxiety components (nodes) for network analyses^26^.

#### Social Interaction Pattern: Liebowitz Social Anxiety Scale (LSAS) & Smartphone Addiction Scale (SAS)

To explore the detailed aftereffect of verbal abuse on the exposed person’s social interaction pattern^67–69^, this study focused on non-confrontational coping for social interaction including the anxiety-fear-avoidance for social situation^56–59^ (measured using the LSAS) and preference for non-face-to-face social interaction behind the smartphone overuse^60–62^ (measured using the SAS).

The LSAS assesses the level of fear/anxiety associated with, and the severity of avoidance of, 24 social situations using a 4-point Likert scale [from 0 = ‘not at all’ to 3 = ‘very much’]^70,71^. Here, we used the self-reporting version of LSAS^72–74^ and derived eight sub-domains that measured fear/anxiety or avoidance for ‘public speaking’ [items 20, 16, 6, 15, and 5], ‘social interaction with strangers’ [items 10, 11, and 12], ‘assertiveness’ [items 21, 22, 24, 18, and 14], and ‘public interaction’ [items 4, 1, 3, 7, 8, and 19]^75^.

The SAS is comprised of 46 items measuring various aspects of smartphone misuse using a 6-point Likert scale [from 1 = ‘not at all’ to 6 = ‘totally agree’]^76^. We derived four SAS sub-domains reflecting 1) daily life disturbance (due to the smartphone overuse), 2) positive anticipation (of emotional reward from smartphone use), 3) withdrawal (from the restriction of smartphone use), and 4) cyberspace-oriented relationships^76^. In the subsequent network analyses, these four sub-domains of SAS served as four nodes reflecting the smartphone-dependent patterns of social interaction that could be associated with experiential avoidance for real-world interaction^60^, difficulty of cognitive control for emotional processing in the middle of face-to-face interactions^77^, as well as loneliness and needs for social belonging combined with lower self-esteem^78–80^.

#### Substance abuse: CAGE questionnaire

The four items of the CAGE questionnaire focus on alcohol misuse, including a need to reduce drinking, perception of annoying criticism, guilty feelings, and use of alcohol as an eye-opener^81,82^. The total score served as the substance-mediated component of the social interaction feature of network analyses.

#### Cognitive style in daily living: Adult ADHD Self-Report Scale (ASRS-v1.1) Symptom Checklist

The ASRS-v.1.1 assesses attention-deficit/hyperactivity disorder (ADHD) symptoms using 18 DSM-IV symptom criteria^83^. We evaluated six Part A ASRS-v1.1 items including: 1) trouble finalising a project; 2) difficulty in organisation; 3) problems remembering appointments or obligations; 4) avoiding commencing tasks requiring a lot of thought; 5) fidgeting or squirming (hands or feet) when sitting for a long time; and, 6) feeling overly active and compelled to do things, as if driven by a motor. For each item, respondents reported the frequencies of such experiences over the prior six months, using five options (never, rarely, sometimes, often, or very often)^84^.

#### Perceived verbal abuse: Verbal Abuse Questionnaire (VAQ)

Lifetime (both earlier and recent) experiences of perceived verbal abuse from parents, supervisors, and peers were measured using the VAQ validated for the Korean college population^85,86^. The VAQ is composed of 15 items covering scolding, yelling, swearing, blaming, insulting, threatening, demeaning, ridiculing, criticising, and belittling; perceived severity was reported using a 9-point Likert scale [from 0 = ‘not at all’ to 8 = ‘everyday’]^85,86^.

### Networks

#### Network 1: Directed acyclic graph of perceived verbal abuse components

**Figure 1:**
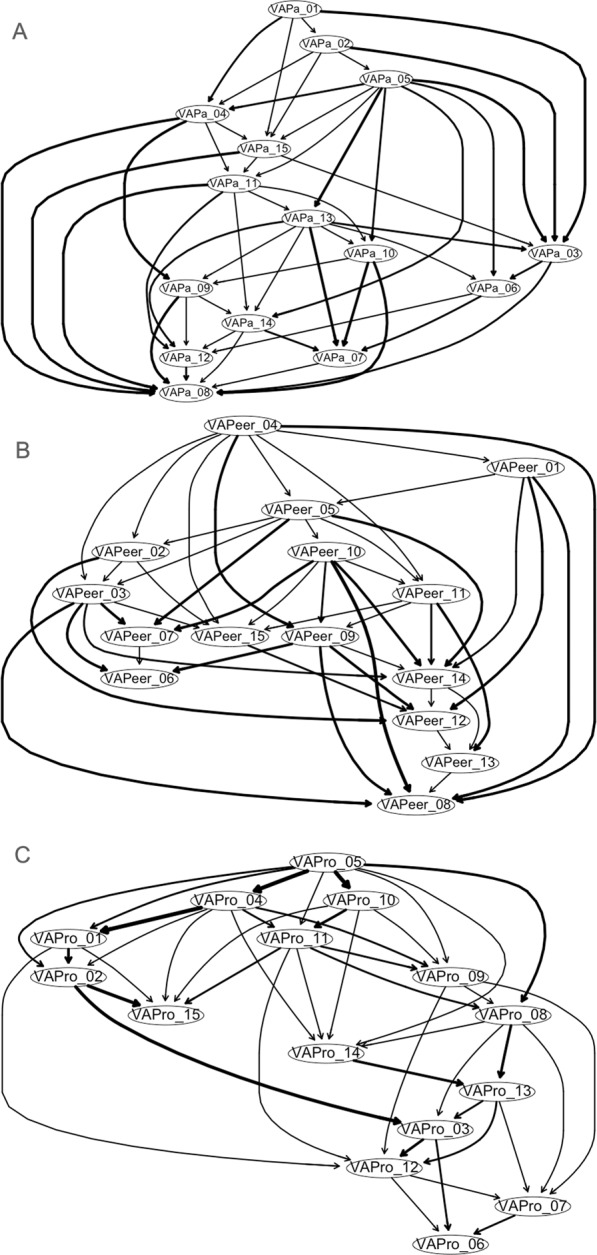
Directed acyclic networks formed using the 15 verbal abuse questionnaire (VAQ) components by (**A**) parents, (**B**) peers, and (**C**) supervisors. The abbreviations are described in Table 1.

**Figure 2:**
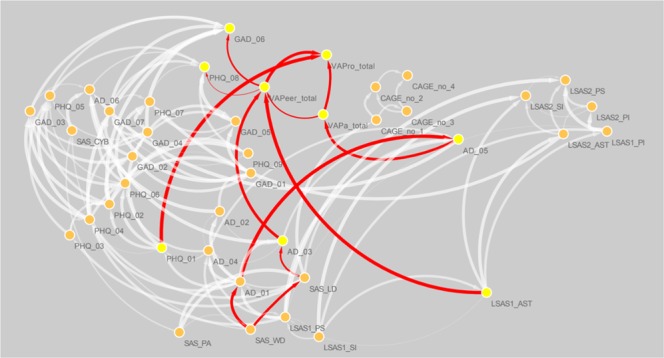
Directed acyclic network comprised of perceived, verbal abuse severity; psychopathology; and social interaction patterns. Perceived, verbal abuse severity components of parents (VAPa_total), peers (VAPeer_total), and supervisors (VAPro_total) are shown, as are six further components directly connected to these components (red arrows) including: 1) fidgeting when sitting for a long time (AD_05); 2) problems remembering appointments or obligations (AD_03); 3) fear of assertiveness (LSAS1_AST); 4) low levels of interest and/or pleasure (PHQ_01); 5) psychomotor change (PHQ_08); and 6) irritability (GAD_06); all are rimmed with yellow circles. The abbreviations are described in Table 1.

To explore the differential patterns of perceived verbal abuse escalation^69,87–93^ according to the source of parents, peers, or supervisors, using the hill-climbing algorithm provided by the R package *bnlearn*^22,24^, we derived three Bayesian networks (each comprised of the 15 items from the VAQ-parents, -peers, or -supervisors, respectively) embodied in DAGs. First, using the bootstrapping function, we extracted 10,000 samples (with replacement) and estimated an optimal network structure for a target goodness-of-fit score (e.g., the Bayesian Information Criterion (BIC) provided by *bnlearn* program for each edge comprising given network; larger absolute BIC value indicate the higher importance of specific edge for the integrity of network in explaining the data)^22^ by randomly adding and removing edges connecting different VAQ items and reversing edge directionality^22^. Notably, to eliminate the possibility of a poor local BIC maximum, we repeated network start/estimation five times; each run included 10 perturbations of edge insertion/deletion or directionality reversal^22^. Only the subset of edges that appeared in at least 85% of the 10,000 networks was retained in the final averaged DAG network^22,94^. Second, the directionality of each edge in the final network was maintained in over 50% of the 10,000 bootstraps; the probability of edge direction reflects edge thickness (thicker or thinner than average) (Figs 1–2**)**^22^. The mean ± S.D. scores for VAQ-parents, VAQ-peers, and VAQ-supervisors were 3.2 ± 7.7 (range 0 to 99), 3.3 ± 7.5 (range 0 to 90), and 2.6 ± 6.4 (range 0 to 78), respectively. All of the procedures for estimation of network 1 were conducted using the modified version of the original R script provided from McNally, *et al*. (2017) and is provided in the supplementary material.

#### Network 2: Directed acyclic graph of perceived verbal abuse severity (VAQ-parents, VAQ-peers, and VAQ-supervisors), psychopathology, and social interaction patterns

Using the procedure described above for *Network 1*, we drew a group-wise DAG to explore the relationships between perceived verbal abuse severity by parents, peers, or supervisors (total scores for VAQ-parents, VAQ-peers, and VAQ-supervisors regardless of the timing of abuse) and depressive mood (the nine items of PHQ-9), anxiety (the seven items of GAD-7), social interaction patterns (the eight LSAS subscores for fear/anxiety or avoidance of public speaking, social interaction with strangers, assertiveness, and public interaction), the four SAS subscores related to smartphone addiction (daily life disturbance, positive anticipation, withdrawal, and cyberspace-oriented relationships), the four items of CAGE exploring problematic alcohol use, and the six items of ASRS-v.1.1 (part A) (difficulty with completion, forgetfulness, procrastination, and hyperactivity)^26,84,95–98^. The mean ± S.Ds. of the 41 items (=nodes) of *Network 2* are listed in Table 1. The network analyses procedures for estimation of network 2 were conducted using the modified version of the original R script provided from McNally, *et al*. (2017) as shown in the supplementary material.

**Table 1:** Clinical characteristics comprising the directed acyclic networks [network 2; grey-colored in column of ‘network 2′] and intra-individual covariance networks [network 3; grey-colored in column of ‘network 3′] of perceived verbal abuse-psychopathology-social interactions (N = 5,616).

Domain	Item	Mean (S.D.)	Network 2	Network 3
Perceived verbal abuse (Verbal abuse questionnaire; VAQ)	[VA*_01] scolded me	0.66 (0.88)		averaged value of three verbal abuse source including parents, peers, and supervisors
	[VA*_02] yelled at me	0.31 (0.61)		
	[VA*_03] swore at me	0.18 (0.48)		
	[VA*_04] blamed me for something	0.30 (0.62)		
	[VA*_05] gave me an insult	0.22 (0.53)		
	[VA*_06] threatened to hit me	0.06 (0.29)		
	[VA*_07] used a nickname that gave me insults	0.10 (0.34)		
	[VA*_08] said I was stupid	0.19 (0.51)		
	[VA*_09] blamed me for what I did not do	0.14 (0.42)		
	[VA*_10] humiliated me in front of others	0.15 (0.42)		
	[VA*_11] criticized me	0.18 (0.48)		
	[VA*_12] yelled at me without reason	0.06 (0.28)		
	[VA*_13] told me that I was useless	0.06 (0.29)		
	[VA*_14] made me feel worthless	0.12 (0.40)		
	[VA*_15] raised one’s voice	0.29 (0.59)		
	[VA*_total] total score of VAQ sourced from parents	3.24 (7.71)		
	[VA*_total] total score of VAQ sourced from peers	3.25 (7.49)		
	[VA*_total] total score of VAQ sourced from supervisors	2.58 (6.42)		
Depressive mood (PHQ-9)	[PHQ_01] low interest or pleasure	0.37 (0.64)		
	[PHQ_02] feeling down, hopeless	0.36 (0.59)		
	[PHQ_03] trouble sleeping	0.49 (0.76)		
	[PHQ_04] tired or little energy	0.64 (0.78)		
	[PHQ_05] poor appetite/overeating	0.34 (0.64)		
	[PHQ_06] guilt	0.22 (0.54)		
	[PHQ_07] trouble concentrating	0.12 (0.41)		
	[PHQ_08] moving slowly/restless	0.06 (0.28)		
	[PHQ_09] suicidal thoughts	0.04 (0.24)		
anxiety (GAD-7)	[GAD_01] nervous, anxious, on edge	0.25 (0.52)		
	[GAD_02] uncontrollable worry	0.23 (0.54)		
	[GAD_03] worry about different things	0.43 (0.70)		
	[GAD_04] trouble relaxing	0.21 (0.52)		
	[GAD_05] restless	0.09 (0.33)		
	[GAD_06] irritable	0.20 (0.47)		
	[GAD_07] afraid something awful might happen	0.09 (0.35)		
social interactions (LSAS)	[LSAS1_PS] fear for public speech	2.81 (3.24)		
	[LSAS2_PS] avoidance from public speech	2.04 (2.67)		
	[LSAS1_SI] fear for social interaction with strangers	1.43 (1.91)		
	[LSAS2_SI] avoidance from social interactions with strangers	1.20 (1.73)		
	[LSAS1_AST] fear of assertiveness	1.66 (2.32)		
	[LSAS2_AST] avoidance from assertiveness	1.45 (2.12)		
	[LSAS1_PI] fear for social interactions at public spaces	1.75 (2.43)		
	[LSAS2_PI] avoidance from social interactions at public spaces	1.96 (2.57)		
social interactions (SAS)	[SAS_LD] daily-life disturbance related to smartphone use	13.90 (6.67)		
	[SAS_PA] positive anticipation for smarphone use	13.36 (5.71)		
	[SAS_WD] withdrawal from smartphone use	11.67 (5.10)		
	[SAS_CYB] cyberspace-oriented relationship	11.47 (4.75)		
substance misuse (CAGE)	[CAGE_no_1] felt you needed to cut down on your drinking	0.16 (0.36)		
	[CAGE_no_2] people annoyed you by criticizing your drinking	0.03 (0.17)		
	[CAGE_no_3] felt guilty about drinking	0.07 (0.26)		
	[CAGE_no_4] felt you needed a drink as an eye-opener	0.01 (0.11)		
	total score [network 3]	0.27 (0.62)		
cognitive styles of task performance (ASRS-v.1.1. part A)	[AD_01] trouble wrapping up final details of a project	0.84 (1.03)		
	[AD_02] difficulty getting things in order for a task requiring organization	0.63 (0.89)		
	[AD_03] problems remembering appointments or obligations	0.74 (0.92)		
	[AD_04] avoiding getting started on a task requiring a lot of thought	1.00 (1.11)		
	[AD_05] fidget or squirm when you have to sit down for a long time	0.96 (1.15)		
	[AD_06] feel overly active and compelled to do things	0.63 (0.95)		

#### Network 3: Intra-individual covariance network of perceived verbal abuse components (regardless of source), psychopathology, and social interaction patterns

We constructed intra-individual covariance networks of clinical features^23^ that reflect the diverse aspects of perceived verbal abuse (the 15 item-level VAQ scores for parental, peer, and supervisor abuse), depressive mood (the nine item-level scores of PHQ-9), anxiety (the seven item-level scores for GAD-7), and social interaction patterns (the eight LSAS subscores, the four SAS subscores, the total CAGE score [the sum of the four values included in network 2]; and the scores for the six items of ASRS-v.1.1 part A) by way of 50 components (=network nodes; Table 1). First, the raw scores were z-transformed using the overall means and standard deviations (derived from the whole group of *N* = 5,616) per clinical feature, as the range of score distribution differs across 50 clinical features comprising the intra-individual covariance network^23^. Second, intra-individual covariances (=network edges) between the 50 clinical features in *k*th participant (*k* = from 1 to 5,616) were calculated using the inverse exponential function that involves the square of the difference between the z-score transformed values of *i*th clinical feature (*z(i*,*k)*, *i* = from 1 to 50) and *j*th clinical feature (*z(j*,*k)*, *j* = from 1 to 50) as below^23^.

$$1/\exp (\{zi,k(-z(j,k)\}r{t}^{2})$$

This formula enables the structural covariance values (=weight of edges in the intra-individual covariance network) to be distributed within the range of 0 and 1, in proportional to the degree of similarity between two different clinical features (=nodes that comprise intra-individual covariance network) per participant. The means ± S.Ds. for the 50 components (nodes) of *Network 3* are listed in Table 1. The Matlab script (=.m file) used for calculation of network 3 is provided as supplementary material.

### Graph theory analyses of Network 3 (an intra-individual covariance network)

To identify the most influential components^99,100^ which mediate the propagation of information as shortcuts in the midst of numerous inter-connected components of verbal abuse-psychopathology-social interaction, the current study estimated a local graph metric named ‘betweenness centrality (=the frequency with which a node is located in the path of a shortcut connecting two different nodes)^99,101^’. The optimal level of *network sparsity* [*K*; defined as the proportion of non-zero edges relative to the total possible number of connections (=*N* × (*N* − 1)/2; *N* = number of nodes) in the network] appropriate for deriving the *betweenness centrality* values were searched under the three criteria^102,103^ of (1) small-world organisation [balanced network for global integration as well as local segregation^103,104^; satisfied when *small-worldness* (σ) > 1]^102,105,106^, (2) modular organisation [network could be subdivided into communities^103,107^; sufficient when *modularity* (Q) >0.3)^102^], and (3) network connectedness [over 80% of the total (=50) nodes were connected to other nodes] in more than 95% of participants (*N* = 5,616)^33^.

Accordingly, four global graph metrics including (a) normalised clustering coefficient [γ; first measured per node using ‘clustering_coef_wu.m^108^’ and then averaged over all 50 nodes in a given network, and finally normalised using the same variable averaged over 10,000 random networks produced from the original network employing ‘randmio_und.m^109^’]; (b) normalised characteristic path length [λ; retrieved from the distance matrix (in which all edge strengths were inverted compared to the original network by way of ‘distance_wei.m’) using ‘charpath.m^106^’, and finally normalised using the same variable calculated from 10,000 random networks alike (a)]; (c) small-worldness [*σ* = *γ*/*λ*]^106^; and, (d) modularity [*Q*; derived by averaging 500 estimations obtained using ‘modularity_und.m’] were calculated. Finally, in the network sparsity ranges of *K* = 0.10–0.21 that satisfied (1) small-world organisation, (2) modular organisation, and (3) network connectedness for more than 95% of participants, the local graph metric of *betweenness centrality* was calculated (using the ‘betweenness_wei.m^99,101^’) at the connection density level of *K* = 0.10; all of the intra-individual covariance networks (network 3) were transformed *thresholding* (by way of ‘threshold_proportional.m^110^’) to be fitted to the network sparsity level to *K* = 0.10 in which only the subset of edges having strongest edges weights (=connectivity strength) remained.

In a scale-free network, the centrality values do not follow a normal distribution. Therefore, after per-participant rank-transformation of the betweenness centrality values using the ‘tiedrank.m’ function of Matlab R2017a, the top 12%-ranked six (=50 nodes × 0.12) nodes in >25% of participants (*n* = 5,616) were defined as hub nodes (in line with previous studies^111,112^ that defined hubs as the top 12% of most consistently ranked nodes for centrality value across the group of participants) for intra-individual covariance networks of ‘verbal abuse-psychopathology-social interaction’. Finally, relationships between the severity of perceived verbal abuse (=total score of VAQ) by parents, peers, or supervisors versus the rank-transformed betweenness centralities of 35 nodes [=15 VAQ components were excluded from the original 50 nodes of network 3, as associations (if any) between verbal abuse severity and betweenness centralities of VAQ nodes would be auto-regressive; *P* < 0.05/35 = 0.001] comprising the intra-individual covariance networks were explored using the Spearman’s rank correlation coefficients (estimated using the ‘corr(‘type’ = ‘Spearman’)’ function of Matlab R2017a). All of the global and local graph metrics were calculated using the Matlab script (=.m file; mainly written with functions of the Brain Connectivity Toolbox^106^) provided as supplementary material.

## Results

### Network 1: A directed acyclic graph of perceived verbal abuse components

These three DAG networks explored the differential cascades^19,22,113^ of perceived verbal abuse escalation^69,87–93^ according to the source of parents (Fig. 1A), peers (Fig. 1B), or supervisors (Fig. 1C). In the DAG reflecting perceived parental verbal abuse (Fig. 1A), verbal aggression commenced with ‘scolded me’ (VAPa_01) and ‘yelled at me’ (VAPa_02). Subsequently, (without any intervention for escalation of parental verbal abuse exposure) the two hub components of ‘insulted me’ (VAPa_05) and ‘told me that I was useless’ (VAPa_13) influenced eight and six other downward DAG network components, respectively; perceived parental verbal abuse finally evolved into ‘said I was stupid’ (VAPa_08).

On the contrary, in the DAG of perceived verbal abuse by peers (Fig. 1B), verbal aggression started as ‘blamed me for something’ (VAPeer_04) and ‘insulted me’ (VAPeer_05) and subsequently propagated into eight and six components, respectively. When additional exposure to other forms of peer-related perceived abuse such as ‘swore at me’ (VAPeer_03; probabilities of affecting the VAPeer_06 90.1% and the VAPeer_08 78.0%), ‘humiliated me in front of others’ (VAPeer_10; probability of affecting the VAPeer_08 93.7%), and ‘blamed me for what I did not do’ (VAPeer_09; probabilities of affecting the VAPeer_06 85.0% and the VAPeer_08 79.0%) were not interrupted or stopped (either by the targeted person or another), finally the victimised person might suffer more severe verbal threats by peers including ‘threatened to hit me (VAPeer_06) and/or ‘said I was stupid’ (VAPeer_08).

In cases of the supervisor-related verbal abuse (Fig. 1C), a form of perceived verbal aggression ‘insulted me’ (VAPro_05) might be the initial component of appearance as shown in the DAG. On the one hand, escalated supervisor-related verbal insults were perceived as ‘raising one’s (=supervisor’s) voice’ (VAPro_15); on the other hand, activation of the verbal abuse supplied by supervisor(s) such as ‘blamed me for something’ (VAPro_04) and ‘criticised me’ (VAPro_11) were shortcuts that activated other six and five downstream components of supervisor-related verbal abuse, respectively. Without efforts to prevent the targeted person from being exposed to ‘swore at me’ (VAPro_03; probability of affecting the VAPro_06 87.8%) or ‘yelled at me without reason’ (VAPro_12; probability of activating the VAPro_06 69.3%) or ‘used a nickname that insulted me’ (VAPro_07; probability of affecting the VAPro_06 55.5%), threat of physical harm such as hitting (VAPro_06) might occur.

### Network 2: Directed relationships of the perceived verbal abuse–psychopathology-social interaction patterns

Next, the patterns of interaction between perceived verbal abuse (VAQ-parents, -peers, and -supervisors scores, evaluated separately) and depressive mood, anxiety, social interactions, alcohol abuse, and inattention-hyperactivity (as estimated by DAGs) were examined (Fig. 2). First, the severity of perceived, parental verbal abuse (VAPa_total) was directly influenced by the intensity of hyperactivity (‘fidgeting or squirming with the hands or feet when you have to sit for a long time’ (AD_05), with a probability of 86.9%. Second, the severity of peer-related, perceived verbal abuse (VAPeer_total) was affected from fear of assertiveness in social situations (LSAS1_AST; probability 96.0%), and inattention and problems remembering appointments or obligations (AD_03; probability of 88.2%), as did perceived, parental verbal abuse (VAPa_total; probability 63.2%). Third, the severity of supervisor-related, perceived verbal abuse (VAPro_total) was accompanied by preceding low interest or pleasure in doing things (PHQ_01; 97.5% probability), as was perceived verbal abuse from other sources (probabilities of 82.4% for ‘VAPa_total’ and 89.8% for ‘VAPeer_total’). Furthermore, the severity of peer-related, perceived verbal abuse affected psychomotor retardation/agitation (PHQ_08; probability 50.1%) and influenced the activation of irritability (GAD_06; probability 65.1%).

### Graph theory analyses for Network 3 (intra-individual covariance network)

**Figure 3:**
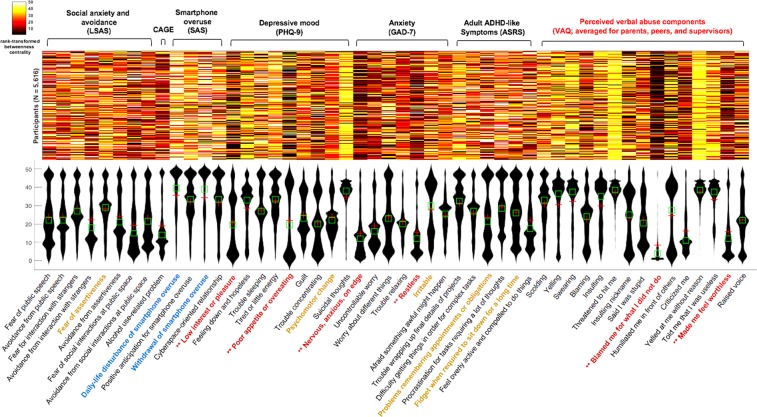
Heatmap (upper) and violin plot (lower) of rank-transformed betweenness centrality values calculated from the intra-individual covariance network (*N* = 5,616) featuring perceived verbal abuse components (averaged over parents, peers, and supervisors), psychopathology, and social interaction patterns. In the x-axis of the violin plot, the six most influential components (hubs; the top 12% nodes for rank-transformed betweenness centrality in >25% of participants at a network sparsity level of *K* = 0.1) are: 1) low-level interest or pleasure; 2) poor appetite or overeating; 3) nervousness; 4) restlessness; 5) blaming oneself for what one has not done; and, 6) feeling worthless, are coloured brown and marked with asterisks.

**Figure 4:**
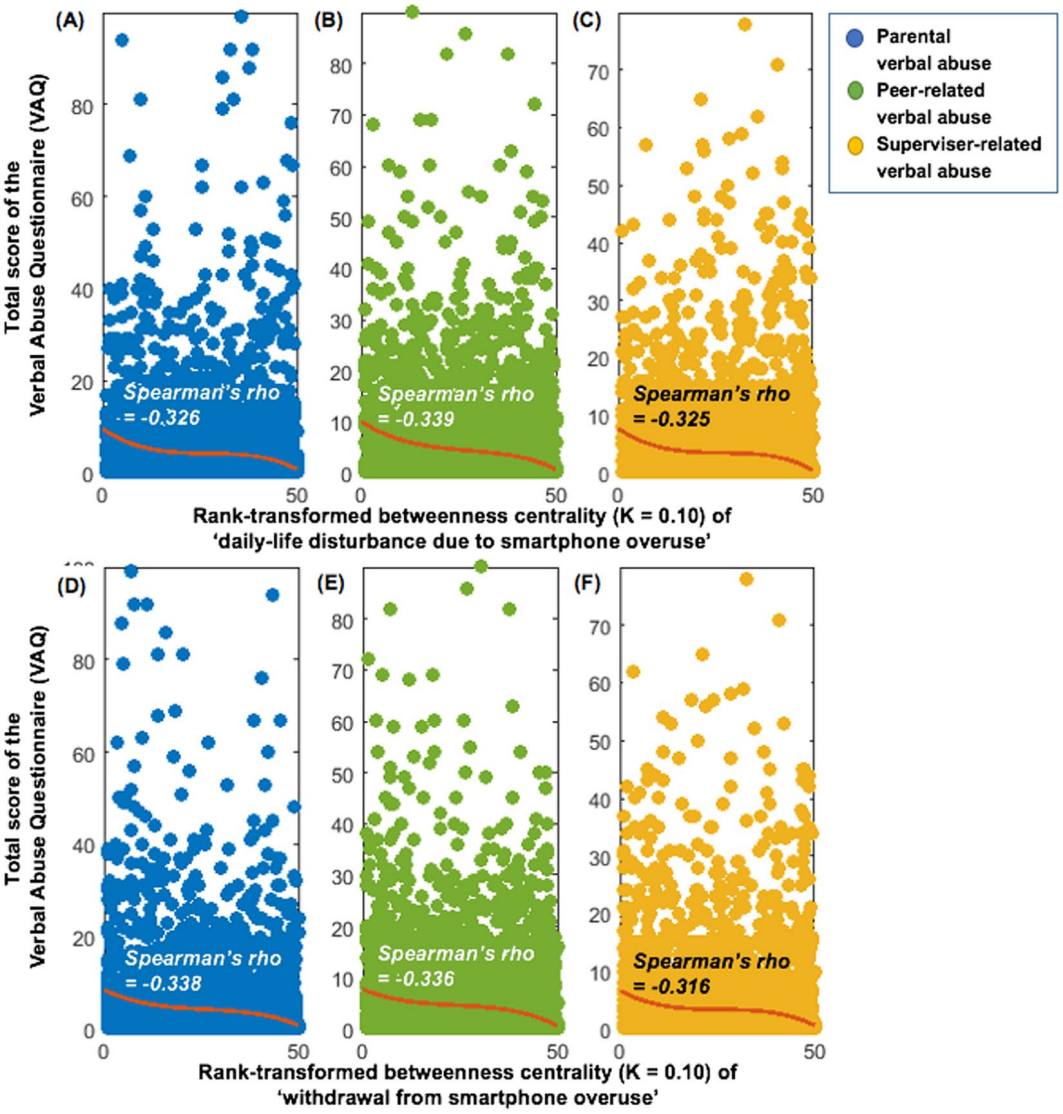
Correlations between total scores on the verbal abuse questionnaire versus the rank-transformed betweenness centralities of daily-life disturbance (**A,B,C**) and withdrawal caused by smartphone overuse (**D,E,F**) calculated from the intra-individual covariance networks (at network sparsity level of *K* = 0.10) of perceived verbal abuse, psychopathology, and social interaction patterns (all *Ps* < 0.001). Brown-colored polynomial curves (degree of polynomial fit = 3) were fitted using the ‘polyfit’ function of Matlab R2017a.

The graph theory approach of the intra-individual covariance network (*N* = 5,616) comprised of perceived verbal abuse (15 item-level VAG scores averaged for the three sources), psychopathology, and social interaction patterns, retrieved six highly influential components (in terms of mediating the propagation of information as shortcuts among the numerous inter-connected components within the network^99,100^; the top 12% ranked variables in terms of rank-transformed betweenness centrality in >25% of participants at a network sparsity of *K* = 10) including low interest or pleasure in doing things (PHQ_01), changed appetite (PHQ_05), nervousness (GAD_01), restlessness (GAD_05), blaming oneself for what one did not do (VAQavg_09), and feeling worthless (VAQavg_14) (Fig. 3**)**. Further, significant relationships between the VAQ total scores and the rank-transformed betweenness centralities of daily life disturbance (Spearman’s *rho* = −0.326, −0.339, −0.325; *P*-values = 2.16 × 10^−139^, 7.26 × 10^−151^, 1.79 × 10^−138;^ for VAQ-parents, -peers, and -supervisors, respectively) and withdrawal caused by smartphone addiction (*rho* = −0.338, −0.336, −0.316; *P-*values = 3.61 × 10^−150^, 3.84 × 10^−148^, 9.33 × 10^−131^ for VAQ-parents, -peers, and -supervisors, respectively), were also evident (Fig. 4A–F**;** significant at *P* < 0.05/35 [number of nodes in network 3 that were not VAQ components] = 0.001; fitted curves with linear regression including polynomial terms of squared and cubic predictors (estimated using the ‘polyfit’ function of Matlab R2017a) also illustrated).

## Discussion

To the best of the authors’ knowledge, this study is the first network-based approach that explored the directed associations among the perceived verbal abuse severity and ‘depressive mood-anxiety-social interaction’ patterns as well as the principal drivers (hubs) in the intra-individual covariance networks of ‘perceived verbal abuse–psychopathology-social interaction’ patterns. The severity of peer-related verbal abuse created a fear of assertiveness and difficulty in remembering appointments or obligations, followed by psychomotor changes and irritability (Fig. 2). Moreover, in addition to the four depressive mood-anxiety components, perceived verbal abuse such as ‘blaming me for what I did not do’ and ‘making me feel worthless’ were hubs connecting differential components of the intra-individual covariance networks (Fig. 3). Of note, the intensity of perceived verbal abuse correlated with the hubness of daily-life disturbance and withdrawal caused by smartphone misuse (Fig. 4). Use of relatively large number of responses (*N* = 5,616) acquired by way of the well-validated self-reporting questionnaires enhanced the power of study results. Further, application of item-level responses or sub-domain scores (comprising the full scales) as nodes in the network-based approach of ‘perceived verbal abuse-psychopathology-social interaction’ improved the resolution of study results so that the most influential psychological items in the network could be profiled as hubs. These study results raise the importance of psychoeducation facilitating nonviolent empathetic communication, training in self-protection from verbal abusers, and timely psychological aid for victimised persons focusing on the dysphoric mood, fear of assertiveness, and smartphone misuse, at least for college population.

### Damaged self-esteem and unjust blame: shortcuts of verbal abuse–psychopathology-social interactions

Verbal abuse that makes one feel worthless (damaged self-esteem) or blames a person for something s/he did not do (unjust blame) were shortcuts connecting different components of the intra-individual covariance networks comprised of the ‘verbal abuse-depressive mood-anxiety-social interaction’ patterns (Fig. 3). Humiliation is a predictor of depression^114^, as is interpersonal sensitivity^115^. When a humiliating experience leads to a fear of further humiliation, a victimised person may become increasingly sensitive to social threats and social anxiety cues^115^. What is worse, poor assertiveness in social situations may create a defensive silence even when verbal abuse is ongoing^116^, as shown here (Fig. 2). In unfamiliar or uneasy social situations, alcohol is used to reduce anxiety^117^ and to supply an emotional reward^118^; however, victimisation by others in social situations may trigger alcohol misuse^119,120^. Further, the perception of severe verbal abuse was associated with increased smartphone addiction more so than other ‘verbal abuse-depressive mood-anxiety-social interaction’ patterns (Fig. 4), in agreement with the results of previous studies suggesting a possible role for smartphone addiction in avoidance of social exclusion-related distress^121^, as a medium for social rehearsal and monitoring^122^, or as an alternative source of a sense of belonging^78^.

### Mood disturbances such as psychomotor changes and irritability: Aftermaths of peer-related verbal abuse

The severity of peer-related verbal abuse affected the extent of psychomotor change and irritability (Fig. 2), escalating depressive mood as previously reported^3^. In other words, post-traumatic anger expression may be either externalised as behavioural aggression-irritability or internalised as depressive mood-anxiety^123^; however, these seemingly contrasting phenotypes might be similarly underpinned neurally via reduced integrity of cingulum bundle white matter in the posterior tail of the left hippocampus^10^. The effect of time cannot be modelled with our cross-sectional data; however, the DAG suggests that causal hypotheses are testable via intervention. In addition to caring for the psychological distress caused by verbal abuse^124^, educational efforts reducing factors preceding such abuse, including the fear of assertiveness and appointment/obligation forgetfulness (Fig. 2), are desirable. It is necessary to enhance assertiveness and communication^125,126^ and to develop behavioural skills enhancing the social-organisational-attentional subdomains^127^.

### Limitations

Our study had certain limitations. First, the precise timing of verbal abuse was not considered. Rather, participants in early adulthood were asked to report the lifelong frequencies of diverse, perceived verbal abuses, regardless of exposure times. Although timepoint resolution was thus absent, and verbal abuse may have decreased over time, we derived more generalisable, abstract patterns of verbal abuse propagation and the relationships thereof with psychological health and social interactions; we did not focus on differential total lifetime exposure, timing, or duration^67,128,129^. Second, the sex ratio of current study population was not balanced (we evaluated 4,498 males (80.1%) and 1,118 females (19.9%)). As a matter of fact, rather than finding the sex-related differences of verbal abuse-psychopathology-social interaction interactions, we combined the study population for network analyses so that both male and female college students integrated to estimate the group-wise DAGs that unveiled the escalating cascades of verbal abuse (network 1) as well as the interacting patterns of verbal abuse-psychopathology-social interaction (network 2). Considering the possible sex-related differences in response to traumatic life events^130,131^, further network-based studies for which larger-sized population with equal proportion of male and female satisfied are required. Third, we did not explore the neural correlates of abuse exposure or interactions between such abuse compared to other psychopathologies. Such brain-based information^12^ would aid our understanding of the biological mechanisms underlying directed^26^ and hierarchical^23^ networks featuring traumatic experiences, psychological health, and social interaction patterns. Fourth, we did not explore the relationship between perceived verbal abuse and neurocognitive abilities^132,133^. Future studies measuring both factors will reveal network-based interactions not only between perceived verbal abuse and psychological health and social interaction patterns (explored in this study) but also between abuse and both cognitive ability and academic achievement.

## Conclusion

We studied 5,616 college students in terms of the propagation patterns of perceived verbal abuse components and found possible directed relationships that hypothetically could propagate from the perceived verbal abuse to the psychomotor changes and irritability^134^. Further, graph theory metrics calculated from the intra-individual covariance networks demonstrated the hubness of some forms of verbal abuse including ‘damaged self-esteem’ and ‘unjust blame’; the hubs served as shortcuts connecting different ‘verbal abuse-depressive mood-anxiety-social interaction’ features. The importance of smartphone misuse-related distress as a shortcut connecting these features was greater for participants who suffered more from perceived verbal abuse. Psychoeducation facilitating nonviolent empathetic communication^135,136^, training in self-protection from verbal abusers^137^, and timely psychological aid for victimised persons focusing on the dysphoric mood, fear of assertiveness, and smartphone misuse^138^, are required.

## Supplementary information

Supplementary Information

## Data Availability

The authors will make materials, data and associated protocols promptly available to readers without undue qualifications in material transfer agreements.
