# Supplementary material for: Verbal Abuse Related to Self-Esteem Damage and Unjust Blame Harms Mental Health and Social Interaction in College Population
Source: Sci Rep. 2019 Apr 4;9:5655. doi: 10.1038/s41598-019-42199-6 (PMC6449380; doi:10.1038/s41598-019-42199-6)
Supplement: Supplementary file 1 — Supplementary Information [file 41598_2019_42199_MOESM1_ESM.docx]

Supplementary Information

Verbal Abuse Related to Self-Esteem Damage and Unjust Blame

Harms Mental Health and Social Interaction in College Population

Je-Yeon Yun^1,2^, Geumsook Shim^3^, Bumseok Jeong^3,4,5,*^

Tables of contents

Supplementary information 1. An R script for the estimation of directed acyclic graphs (DAGs) named ‘Network 1& Network 2’

Supplementary information 2. A Matlab script for the construction of Network 3 & calculation of global-local graph metrics for Network 3

Supplementary information 1. An R script for the estimation of directed acyclic graphs (DAGs) named ‘Network 1 & Network 2’

## The network analyses procedures for estimation of networks 1 & 2 were conducted

## using the R script shown below: a modified version of the original R script provided from

## McNally, et al. (2017) entitled as

## "Comorbid obsessive-compulsive disorder and depression: A Bayesian network approach".

## ---- required packages

## Rgraphviz is on BioConductor, the remaining packages on CRAN

## source("http://bioconductor.org/biocLite.R")

## biocLite("Rgraphviz")

source("https://bioconductor.org/biocLite.R")

biocLite("Rgraphviz")

require("psych")

require("qgraph")

require("bootnet")

require("bnlearn") ## Bayesian network package

require("Rgraphviz") ## used for visualizing Bayesian networks

require("readr")

library("psych")

library("qgraph")

library("bootnet")

library("bnlearn")

library("Rgraphviz")

library("readr")

## ------------------------ data import and data manipulation -------------------------------

datRogers <- read.csv("TOTAL_VAPa_20180725.csv") ## import data: could be changed for file name

head(datRogers)

dim(datRogers)

netdata <- as.data.frame(apply(datRogers, 2, as.numeric)) ## convert to numerics

## ---------------------------------- Bayesian network ---------------------------------

## Fit a first Bayesian network, based on 50 random re-starts and 100 perturbations for each re-start.

set.seed(123)

fitBN1 <- hc(netdata, restart = 50, perturb = 100) ## hc gives directed graph

fitBN1

bnlearn::score(fitBN1, data = netdata) ## global network score

astr <- arc.strength(fitBN1, netdata, "bic-g") ## connection strength

astr[order(astr[,3]), ] ## sorted edge strength from strongest to weakest

strength.plot(fitBN1, astr, shape = "ellipse")

## Now we stabilize the network across multiple samples through bootstrapping:

## Learn 10,000 network structures

## (might take several hours; we keep the number of restarts and perturbations considerably low)

set.seed(123)

bootnet <- boot.strength(netdata, R = 10000, algorithm = "hc", algorithm.args = list(restart = 5, perturb = 10), debug = TRUE)

head(bootnet)

## strength: connection strength,

## e.g. 0.86 means that this connection appears in 86% of the fitted networks.

## direction: probability of the direction,

## e.g. 0.57 means that in 57% of the fitted networks the connection goes in

## the direction depicted in the graph.

## filter the ones with a strength larger than 0.85 and a direction probability > 0.5

bootnet[bootnet$strength > 0.85 & bootnet$direction > 0.5, ]

## build the average network using a 0.85 threshold (Sachs et al., 2005, Science)

avgnet1 <- averaged.network(bootnet, threshold = 0.85)

avgnet1

bnlearn::score(avgnet1, data = netdata)

astr1 <- arc.strength(avgnet1, netdata, "bic-g") ## compute edge strengths

strength.plot(avgnet1, astr1, shape = "ellipse")

## FINAL DAG: use net1 threshold, edge strengths are determined by direction probability

## thick arrows indicate high directional probabilties, thin arrows low directional probabilities

boottab <- bootnet[bootnet$strength > 0.85 & bootnet$direction > 0.5, ]

boottab

astr4 <- boottab ## table with direction probabilities

astr4$strength <- astr4$direction ## use the direction probabilities for edge width

strength.plot(avgnet1, astr4, shape = "ellipse")

Supplementary information 2. A Matlab script for the construction of Network 3 & calculation of global-local graph metrics for Network 3

%% 1. calculate M and SD per variable

for i=1:50

datRogers_M(i,1)=mean(datRogers{:,i});

datRogers_SD(i,1)=std2(datRogers{:,i});

end

%% 2. z-transform the raw scores

for i=1:50

for j=1:5616

z_transform(j,i)=(datRogers{j,i}-datRogers_M(i,1))./(datRogers_SD(i,1));

end

end

%% 3. calculation of network 3

for j=1:5616

variability_per_indi{j,1}=zeros(50,50);

for i=1:50

for k=(i+1):50

variability_per_indi{j,1}(i,k)=1./exp((z_transform(j,i)-z_transform(j,k))^2);

variability_per_indi{j,1}(k,i)=1./exp((z_transform(j,i)-z_transform(j,k))^2);

end

end

fprintf('j=%d\n',j)

end

%%

%% 4.calculate global & regional network characteristics: weighted ver (using 2013 ver of BCT)

clear A

clear C

clear D

for p=1:5616

W=variability_per_indi{p,1};

for i=5:30

A=threshold_proportional(W,i./100);

% clustering coefficient

C = mean(clustering_coef_wu(A)); % A=binary undirected connection matrix; C = clustering coefficient vector

% characteristic path length

D = distance_wei(1./A);

lambda = charpath(D); % D=distance matrix; lambda=characteristic path length

% global efficiency

Eglob = efficiency_wei(A); % Eglob=gloval efficiency(scalar)

% normalize features above using rand networks

for k=1:1000

R = randmio_und(A,20);

C_rand(k,1) = mean(clustering_coef_wu(R));

D_rand = distance_wei(1./R);

lambda_rand(k,1) = charpath(D_rand);

Eglob_rand(k,1) = efficiency_wei(R);

end

global_char_wei.gamma(p,i-4)=C./mean(C_rand);

global_char_wei.lambda(p,i-4)=lambda./mean(lambda_rand);

global_char_wei.GE(p,i-4)=Eglob./mean(Eglob_rand);

global_char_wei.sigma(p,i-4)=(C./mean(C_rand))./(lambda./mean(lambda_rand));

clear C_rand

clear lambda_rand

clear Eglob_rand

% modularity

for k=1:500

[Ci Q(k,1)] = modularity_und(A);

end

global_char_wei.Q(p,i)=mode(Q);

clear Q

% betweenness centrality

regional_char_wei.rank_transformed_betweenness{p,1}(i-4,:) = tiedrank(transpose(1./betweenness_wei(1./A)));

fprintf('p=%d,i=%d\n',p,i)

clear A

clear C

clear D

end

save('graph_wei_today.mat','global_char_wei','regional_char_wei')

end
